# RASAL2 promotes tumor progression through LATS2/YAP1 axis of hippo signaling pathway in colorectal cancer

**DOI:** 10.1186/s12943-018-0853-6

**Published:** 2018-07-23

**Authors:** Yi Pan, Joanna Hung Man Tong, Raymond Wai Ming Lung, Wei Kang, Johnny Sheung Him Kwan, Wing Po Chak, Ka Yee Tin, Lau Ying Chung, Feng Wu, Simon Siu Man Ng, Tony Wing Chung Mak, Jun Yu, Kwok Wai Lo, Anthony Wing Hung Chan, Ka Fai To

**Affiliations:** 1 Department of Anatomical and Cellular Pathology, State Key Laboratory in Oncology in South China, Prince of Wales Hospital, The Chinese University of Hong Kong, 30-32 Ngan Shing Street, Shatin, NT, Hong Kong, SAR China; 2 Li Ka Shing Institute of Health Science, Sir Y.K. Pao Cancer Center, The Chinese University of Hong Kong, Hong Kong, SAR China; 3 Institute of Digestive Disease, Partner State Key Laboratory of Digestive Disease, The Chinese University of Hong Kong, Hong Kong, SAR China; 4 Division of Colorectal Surgery, Department of Surgery, Prince of Wales Hospital, The Chinese University of Hong Kong, Hong Kong, SAR China; 5 Department of Medicine and Therapeutics, Prince of Wales Hospital, The Chinese University of Hong Kong, Hong Kong, SAR China

**Keywords:** RASAL2, Hippo, LATS2, YAP1, Colorectal cancer

## Abstract

**Background:**

Patients with colorectal cancer (CRC) have a high incidence of regional and distant metastases. Although metastasis is the main cause of CRC-related death, its molecular mechanisms remain largely unknown.

**Methods:**

Using array-CGH and expression microarray analyses, changes in DNA copy number and mRNA expression levels were investigated in human CRC samples. The mRNA expression level of RASAL2 was validated by qRT-PCR, and the protein expression was evaluated by western blot as well as immunohistochemistry in CRC cell lines and primary tumors. The functional role of RASAL2 in CRC was determined by MTT proliferation assay, monolayer and soft agar colony formation assays, cell cycle analysis, cell invasion and migration and in vivo study through siRNA/shRNA mediated knockdown and overexpression assays. Identification of RASAL2 involved in hippo pathway was achieved by expression microarray screening, double immunofluorescence staining and co-immunoprecipitation assays.

**Results:**

Integrated genomic analysis identified copy number gains and upregulation of RASAL2 in metastatic CRC. RASAL2 encodes a RAS-GTPase-activating protein (RAS-GAP) and showed increased expression in CRC cell lines and clinical specimens. Higher RASAL2 expression was significantly correlated with lymph node involvement and distant metastasis in CRC patients. Moreover, we found that RASAL2 serves as an independent prognostic marker of overall survival in CRC patients. In vitro and in vivo functional studies revealed that RASAL2 promoted tumor progression in both *KRAS/NRAS* mutant and wild-type CRC cells. Knockdown of RASAL2 promoted YAP1 phosphorylation, cytoplasm retention and ubiquitination, therefore activating the hippo pathway through the LATS2/YAP1 axis.

**Conclusions:**

Our findings demonstrated the roles of RASAL2 in CRC tumorigenesis as well as metastasis, and RASAL2 exerts its oncogenic property through LATS2/YAP1 axis of hippo signaling pathway in CRC.

**Electronic supplementary material:**

The online version of this article (10.1186/s12943-018-0853-6) contains supplementary material, which is available to authorized users.

## Background

Despite an increase in its public awareness, colorectal cancer (CRC) remains one of the most common cancers and the second leading cause of cancer-related death globally [1, 2]. Some genes involved in cell growth, survival, adhesion, invasion and angiogenesis have already been suggested to contribute to tumor progression in CRC [3]. However, a full understanding of the key elements dictating the oncogenic phenotype as well as the molecular events supporting tumor progression undoubtedly helps discover new drugs and ways to prevent CRC.

The hippo signaling pathway is a highly conserved tumor suppressor pathway best known for its role in organ size regulation. The core kinase components MST1/2, WW45, LATS1/2, and MOB1 phosphorylate the downstream transcriptional co-activators YAP and TAZ [4]. Growing evidence suggests that hippo pathway dysregulation is associated with CRC development [5–8]. The gene that has received the most attention in the literature is YAP1, which was found to be involved in the development and progression of CRC [5, 6, 9, 10]. The gene was reported to be overexpressed in 52.5% (73/139) of CRC, and the YAP1 protein was predominantly localized to the nucleus [11]. In addition, YAP1 was considered as a prognostic factor for overall survival in CRC patients [11].

In the present study, using high-resolution array comparative genomic hybridization (aCGH) and gene expression microarray, we identified genes that were differentially expressed among primary tumor, metastasis as well as normal colon mucosa samples, one of which was RASAL2. RASAL2 is a member of RAS GTPase-activating protein family (RAS GAP) that negatively regulates RAS by catalyzing the hydrolysis of RAS-GTP to RAS-GDP. The gene may exhibit pro-tumorigenic or anti-tumorigenic behavior depending on the cell context and/or type of stimulus in human cancers [12–19]. Therefore, the functional role of RASAL2 in CRC was still unclear. Here, we studied the genetic alterations, clinical implications and biological effects of RASAL2 in CRC. In addition, we dissected the mechanistic role of the gene in LATS2/YAP1 activation, and the results suggested that RASAL2 promoted tumorigenesis and metastasis via activation of the hippo pathway through the LATS2/YAP1 axis.

## Methods

### CRC tissues

Primary and metastatic tumor and normal frozen tissues from 8 patients who underwent colectomy and/or metastasectomy at the Prince of Wales Hospital, Hong Kong were used for genomic studies (Additional file 1: Table S1). Archival formalin-fixed paraffin-embedded (FFPE) tissue specimens from 208 CRC patients who underwent colectomy at the same hospital between 1995 and 2014 were retrieved. All specimens were reviewed by an expert gastrointestinal pathologist (KFT) to confirm histological diagnosis and tumor cell content. Clinicopathological information was retrieved from the hospital database last updated in December 2015. The study was approved by Committees for Clinical Research Ethics of Joint Chinese University of Hong Kong-New Territories East Cluster.

### Genomic studies

Microarray-based comparative genomic hybridization (array-CGH) analysis was performed using SurePrint G3 Human CGH Microarray Kit, 1 × 1 M (Agilent Technologies, Santa Clara, CA, USA). Gene expression array analysis was carried out by Macrogen (Seoul, South Korea).

### RNA extraction and quantitative real-time polymerase chain reaction (qRT-PCR)

Total RNA was isolated according to the protocol of TRIzol reagent (Thermo Fisher Scientific, Waltham, MA, USA). Reverse transcription (RT) to synthesize complementary DNA (cDNA) was performed using High-Capacity cDNA Reverse Transcription Kit (Thermo Fisher Scientific). The aliquots of cDNA were amplified using SYBR Green Master Mix (Thermo Fisher Scientific). β-actin was used as endogenous control. The RASAL2 and β-actin primers (Additional file 1: Table S2) were designed using Primer3web [20, 21].

### Immunohistochemistry and scoring

Immunohistochemistry (IHC) was performed on 5-μm sections from tissue microarray (TMA) blocks stained using anti-RASAL2 antibodies (ab121578, 1:400, Abcam, Cambridge, MA, USA) and anti-YAP1 antibodies (ab52771, 1:500, Abcam). The protein expression on the TMA slides was assessed using the histoscore (H-score) method.

### Protein extraction and western blot analysis

Protein was extracted in ice-cold RIPA lysis buffer containing complete protease inhibitor cocktail tablets (Roche, Rotkreuz, Switzerland). RASAL2 was detected with a polyclonal anti- RASAL2 antibody (1:250, ab121578, Abcam). Anti-YAP1 antibody (1:20000, ab52771) was provided by Abcam. Other primary antibodies were from Cell Signaling (Danvers, MA, USA), including antibodies to LATS2 (1:1000, #5888S), phospho-YAP1 (S127) (1:1000, #4911) and Cyclin D1 (1:1000, #2926). β-actin (1:100000, A5441, Sigma-Aldrich, St. Louis, MO, USA) expression was used as an equal loading control. The secondary antibodies were anti-Mouse IgG-HRP (1:15000, 00049039, Dako, Agilent Technologies, Santa Clara, CA, USA) and anti-Rabbit IgG-HRP (1:5000, 00028856, Dako).

### Cell culture, siRNAs /shRNAs and DNA plasmid

Immortalized human normal colon epithelial cells NCM460 [22] and human CRC cell lines Caco2, CL-14, DLD-1, HCT 116, HT-29, LoVo, LS 180, SW480 and SW620 were used in this study. The NCM460 cell line was obtained from Dr. Jun YU [23, 24] (Department of Medicine & Therapeutics, The Chinese University of Hong Kong), who purchased from INCELL Corporation LLC (San Antonio, TX, USA). The cell line was cultured using M3BaseTM medium (INCELL) with 10% FBS. Caco2, DLD-1, HCT 116, HT-29, LoVo, LS 180, SW480 and SW620 were purchased from American Type Culture Collection (ATCC, Manassas, VA, USA). CL-14 cell line was obtained from Deutsche Sam lung von Mikroorganismen und Zellkulturen (Braunschweig, Germany). Constructs of ectopic RASAL2 expression, siRNAs, and shRNAs for RASAL2 knockdown can be found in the Additional file 1: Tables S2 and S3.

### In vitro functional studies

Cell viability was measured using 3-(4,5-dimethylthiazol-2-yl)-2,5- diphenylte-trazolium bromide (MTT, Sigma-Aldrich) assay. For colony formation, transfected cells were cultured for 14 to 21 days, and then fixed with methanol and stained with 0.5% crystal violet. For soft agar assay, three milliliters of the transfected cell-agarose mixture were overlaid onto the base agarose. The plates were incubated for 3–4 weeks, and colonies were stained with 1 mg/ml p-iodonitrotetrazolium violet (INT, Sigma-Aldrich) for visualization. For cell cycle analysis, transfected cells were analyzed in a FACS Calibur Flow Cytometer and data was processed with CellQuest (BD Biosciences). Cell invasion and migration assays were analyzed using Biocoat Matrigel Invasion Chambers (Corning, Bedford, MA, USA) and sterilized transwell insert chambers (Corning), respectively. All experiments were performed in duplicate wells in *n* = 3 independent experiments and results were presented as mean ± SD.

### In vivo tumorigenic assays

1 × 10^6^ transfected CRC cells suspended in 100 μl PBS were injected subcutaneously into the dorsal region of anaesthetized nude mice (5 mice/construct, control in left and treatment in right). When tumor was formed, tumor diameter was recorded every three days for three consecutive weeks. At the end of investigation, mice were sacrificed and xenografts were then collected for diameter and weight measurements. All animal handling and experimental procedures were approved by the Department of Health of Hong Kong and the CUHK.

### Double immunofluorescence staining

Cells grown on coverslips were fixed with 4% paraformaldehyde, permeated with 0.3% Triton X-100 and blocked with 1% BSA. Then cells were in turn incubated with a mixture of two primary antibodies (rabbit anti-YAP1, 1:500 and mouse anti-β-actin, 1:1000) at 4 °C overnight, as well as a mixture of secondary antibodies (Alexa Fluor 488 and 594, 1:2000, Thermo Fisher Scientific) in the dark for 1 h. Nuclei were counterstained by 4′,6-diamidino-2-phenylindole (DAPI, Thermo Fisher Scientific). Images were captured using an Axio Imager2 microscope (Carl Zeiss, Germany).

### Co-immunoprecipitation assay

Cells were transiently co-transfected with siRASAL2 and HA-Ubiquitin plasmids [25]. Then the cells were kept in 20 μM MG132 (Calbiochem, Millipore, Billerica, MA, USA) for 4 h before protein collection. 500 μg cell lysate was incubated with a mixture of 50 μl Protein G bead and 5 μg conjugated antibody to YAP1 at 4 °C overnight with rotation. Finally, samples were loaded onto a SDS-PAGE gel for western blot analysis. Ubiquitination was detected with anti-HA-tag antibody (1:7000, ab9110, Abcam).

### Half maximal inhibitory concentration (IC50) assay of verteporfin

The transfected cells were seeded into 96 well plates and starved for 24 h. Fresh medium with 2-fold diluted drug verteporfin concentration was added to each well. MTT assay was performed after incubation for 24 h.

### Statistical analysis

All statistical analyses were performed in IBM SPSS Statistics (Version 19.0, IBM, Armonk, NY, USA). The expression levels between tumors and matched non-tumor tissues was analyzed using a paired Student’s t-test. An independent Student’s t-test was used to compare the mean expression value of any two pre-selected groups. A Pearson χ2 test was used to assess the association of target gene expression with the clinicopathological parameters. Pearson’s correlation coefficients were used to measure the correlation between two indices in clinical samples. The Kaplan-Meier method was used to plot the survival curves and the log-rank test was used to assess survival difference. Cox proportional hazard regression model was used to analyze independent prognostic factors. Two-tailed *P*-values of < 0.05 and those of < 0.01 were considered as statistically significant and highly statistically significant, respectively.

## Results

### RASAL2 copy number gains and overexpression were observed in CRC

**Fig. 1:**
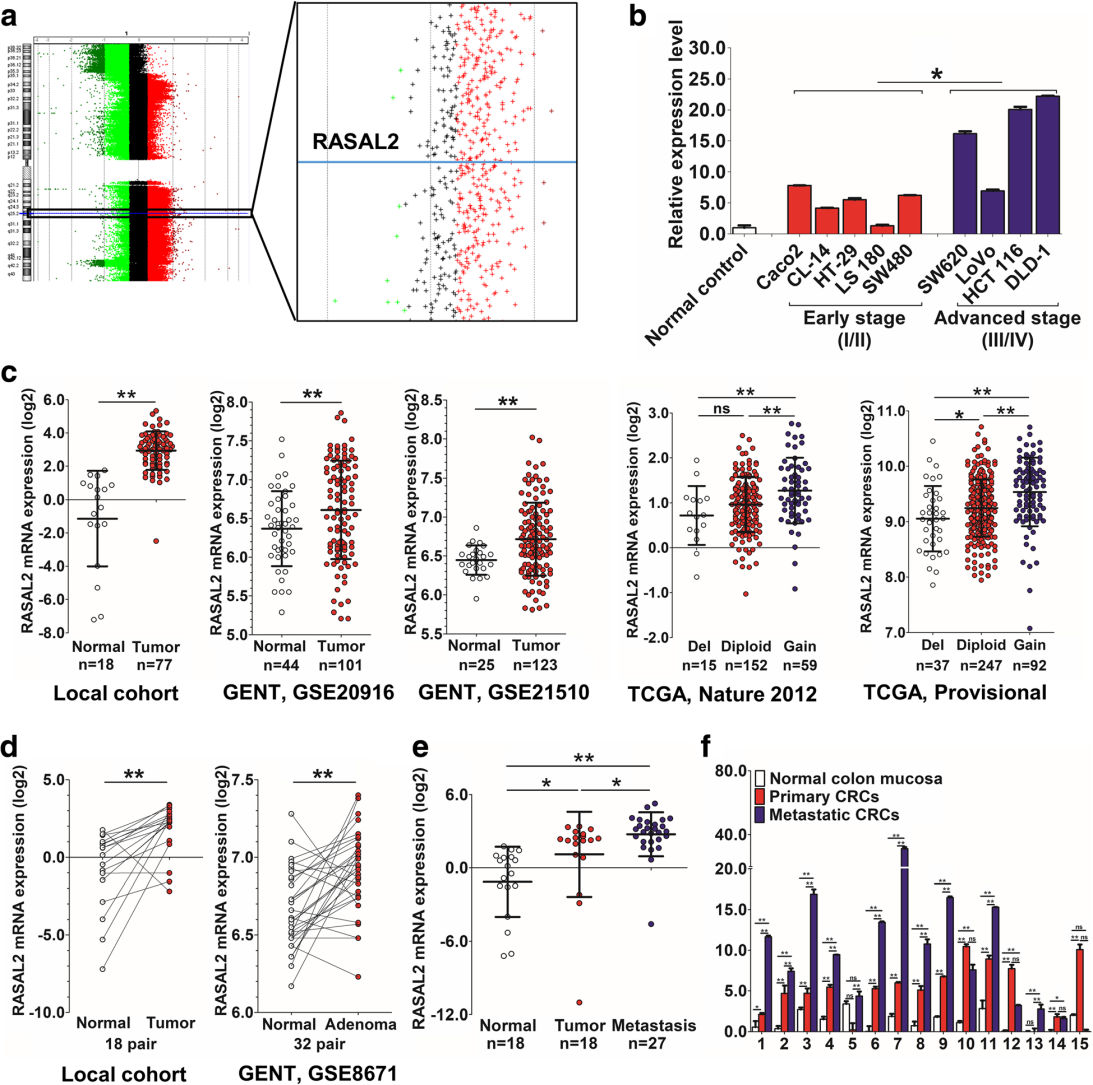
Copy number changes and mRNA expression of RASAL2 in CRC. **a** RASAL2, located on chromosome 1q, is one of the candidate genes showing increased copy numbers in the metastatic tumors in array-CGH. **b** RASAL2 mRNA expression was higher in CRC cell lines than in normal colons, and significantly higher in advanced stage cell lines than in early stage ones. **c** Upregulation of RASAL2 mRNA was found in primary tumors in local cohorts, GENT data and TCGA cohorts. **d** Higher RASAL2 mRNA expression was observed in primary tumors than their matched normal counterparts in our local cohort and GENT data. **e** RASAL2 mRNA expression was the highest in metastatic tumors, followed by primary tumors and normal colon mucosa in local cohort. **f** Out of 15 paired primary and metastatic tumor samples, 12 (80%) had a higher RASAL2 mRNA expression level in metastatic tumors than their primary counterparts. (*, *P* < 0.05; **, *P* < 0.01)

By high-resolution microarray-based comparative genomic hybridization (array-CGH), we found recurrent gains of chromosome 1q in 5 out of 8 metastatic CRC patients (Additional file 1: Table S4). We further employed gene expression microarrays to identify genes differentially expressed among primary tumor, metastasis and normal colon mucosa samples (Additional file 2: Figure S1), one of which was RASAL2. RASAL2 was overexpressed in primary and metastatic tumor samples in both array-CGH (Fig. 1a) and expression microarray analyses (Additional file 1: Table S5).

We examined the mRNA expression level of RASAL2 in a panel of CRC cell lines including 5 lines established from early stage CRC (LS 180, CL-14, HT-29, SW480 and Caco-2) and 4 lines from advanced stage CRC (LoVo, SW620, HCT 116 and DLD-1). RASAL2 mRNA expression was higher in CRC cell lines than in normal colons, and significantly higher in advanced stage cell lines [American joint committee on cancer (AJCC) stage III and IV] than in early stage ones (AJCC stage I and II) (*P* < 0.05, Fig. 1b).

We evaluated RASAL2 mRNA expression in multiple groups of primary tumors and normal colon mucosa. Upregulation of RASAL2 mRNA in CRC was validated independently in the Hong Kong local cohort (*P* < 0.01), Gene Expression across Normal and Tumor tissue (GENT) [26] (GSE20916 [27], *P* < 0.01 and GSE21510 [28], *P* < 0.01) and The Cancer Genome Atlas (TCGA) [29, 30] (*P* < 0.05) (Fig. 1c). We further assessed RASAL2 mRNA expression in 18 pairs of primary tumor and matched adjacent normal tissues. The mean RASAL2 expression was significantly higher in primary tumors compared with their matched adjacent normal colon mucosa (*P* < 0.01, Fig. 1d). A similar trend was observed in 32 paired primary tumor and normal samples in GENT data [26] (GSE8671 [31], Fig. 1d, *P* < 0.01). We also examined RASAL2 mRNA expression in 27 metastatic CRC specimens from patients with liver metastasis underwent hepatic resection. Metastatic tumors showed the highest RASAL2 mRNA expression (*P* < 0.05 vs. primary tumors), followed by primary tumors (*P* < 0.05 vs. normal), and normal colon mucosa (*P* < 0.01 vs. metastasis, Fig. 1e). Out of 15 cases with both primary and metastatic tumor samples, 12 (80%) had a higher RASAL2 mRNA expression level in metastatic tumors than in their primary counterparts (*P* < 0.05, Fig. 1f).

### Upregulation of RASAL2 protein expression was associated with poor prognosis and metastasis in CRC

**Fig. 2:**
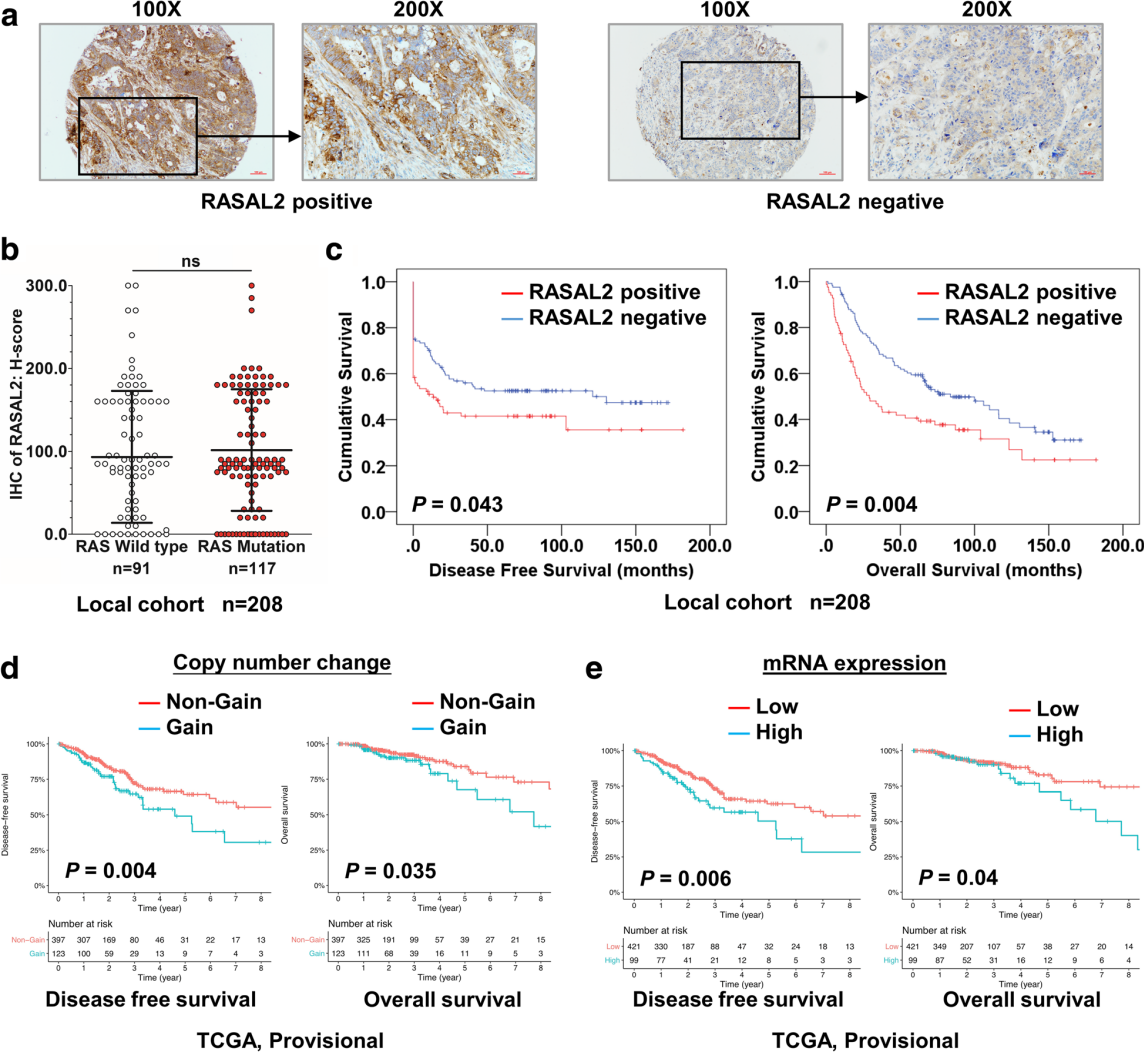
Upregulation of RASAL2 is associated with poor prognosis and metastasis of CRC. **a** Representative IHC images showing positive (H-score > 100) and negative (H-score ≤ 100) RASAL2 expression in CRC. **b** The distribution of RASAL2 histoscore in RAS wild-type and mutant CRC samples. **c** RASAL2 overexpression had significantly shorter disease-free survival and overall survival in a cohort of 208 CRC patients. **d**-**e** Copy number gain (**d**) and upregulated mRNA levels (**e**) of RASAL2 showed aggressive disease-free survival and overall survival in TCGA CRC cohort

We evaluated RASAL2 protein expression in primary and metastatic colorectal tumors as well as normal tissues by IHC on tissue microarrays. RASAL2 proteins were predominantly cytoplasmic in the tumor cells. Figure 2a show representative IHC positive (H-score > 100) and negative (H-score ≤ 100) images. Upregulation of RASAL2 protein expression was observed in 40.4% (84/208) of primary CRCs. Moreover, there was no significant difference in RASAL2 protein expression level between RAS mutant and RAS wild-type CRC cases (Fig. 2b). RASAL2 overexpression was also independent of *KRAS/NRAS* mutation status as well as gene expression subtypes in the TCGA data (Additional file 2: Figure S2). As shown in Additional file 1: Table S6, overexpression of RASAL2 was positively associated with advanced TNM stages (*P* = 0.018), including lymphatic metastasis (*P* = 0.013) and distant metastasis (*P* = 0.007). Kaplan-Meier survival analysis showed that CRC patients with RASAL2 positive expression had significantly shorter disease-free survival and overall survival compared with those without (*P* = 0.043 and 0.004, Fig. 2c). Multivariate analysis by Cox’s proportional hazards regression model revealed that RASAL2 positive expression is an independent prognostic factor for shorter overall survival in CRC (relative risk (RR): 1.561; 95% CI: 1.074 ~ 2.269; *P* = 0.02, Additional file 1: Table S7). We validated the prognostic significance of RASAL2 in TCGA (*n* = 520), Kaplan-Meier survival analysis also showed that copy number gain of RASAL2 and higher RASAL2 mRNA expression was correlated with aggressive disease-free survival (Fig. 2d) and overall survival in CRC patients (Fig. 2e).

### RASAL2 knockdown exerted anti-oncogenic role both in vitro and in vivo

**Fig. 3:**
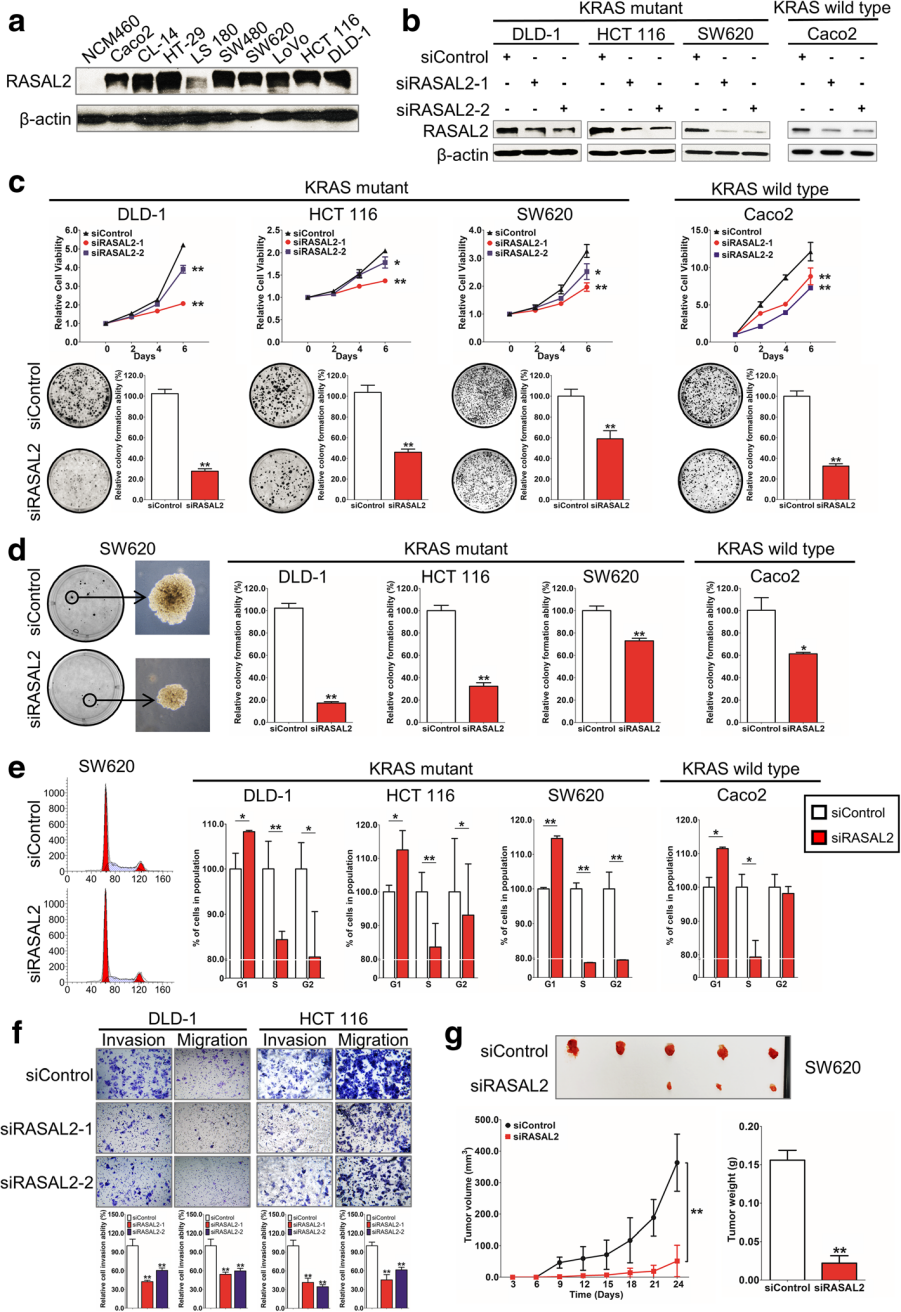
RASAL2 silencing exhibits inhibitory effect in CRC. **a** RASAL2 endogenous expression in CRC cell lines by western blot. β-actin was used as a loading control. **b** siControl or siRASAL2 was transfected into DLD-1, HCT 116 and SW620 and Caco2 cells. The downregulated RASAL2 was confirmed by western blot. **c** siRASAL2 significantly reduced the cell proliferation and anchorage-dependent growth with MTT assays and colony formation assays in *KRAS/NRAS* wild-type and mutant CRC cells. **d** Reduced anchorage-independent growth in RASAL2 knockdown cells using soft agar assays. **e** Flow cytometry analysis on cell cycle in RASAL2 knockdown cells. Representative figure (Left) and percentage of cells in each of the G1, S and G2 phases (Right). **f** Knockdown of RASAL2 by two different siRNAs suppressed cell invasion and migration in DLD-1 and HCT 116 cell lines by cell invasion and migration assays. **g** Tumors isolated from nude mice at the end of investigation (Upper: siControl; Lower: siRASAL2); Tumor growth was summarized using a line chart, while mean tumor weights in the siControl and siRASAL2 groups were shown in the histogram. (*, *P* < 0.05; **, *P* < 0.01, Student’s t-test, *n* = 3 independent experiments)

Figure 3a shows RASAL2 protein expression in normal colon and CRC cell lines by western blot. RASAL2 was frequently upregulated in CRC, suggesting its oncogenic role. Its expression could be silenced with siRNA in the CRC cell lines. Successful knockdown of RASAL2 in *KRAS/NRAS* wild-type (DLD-1, HCT 116 and SW620) and mutant (Caco2) cell lines were confirmed by western blot (Fig. 3b). Using MTT cell proliferation and colony formation assays, a significant decrease in cell proliferation rate and anchorage dependent growth were observed in both *KRAS/NRAS* wild-type and mutant cell lines (Fig. 3c). Using soft agar assay, reduced anchorage-independent growth ability was seen in RASAL-knockdown cells (Fig. 3d). Flow cytometry showed that siRASAL2 inhibited cell growth through inducing cell cycle arrest at G1 phase in the CRC cell lines (Fig. 3e). Western blot analysis revealed that siRASAL2 suppressed the G1-S transition promoter cyclin D1, confirming that siRASAL2 blocked the cell cycle at the G1/S checkpoint (Additional file 2: Figure S3). Next, we examined the effect of RASAL2 knockdown on cell motility as well as invasiveness. We observed that knockdown of RASAL2 by two different siRNAs suppressed cell invasion and migration in DLD-1 and HCT 116 cell lines using Matrigel transwell invasion and cell migration assays, respectively (Fig. 3f). SW620 and Caco2 cells were not studied for the two assays due to endogenous weakness for invasion and migration abilities. To evaluate the effect of siRASAL2 in vivo, early passage of SW620 cells transfected with siControl or siRASAL2 were subcutaneously inoculated on the left or right flank of the anaesthetized mice. The siRASAL2 group formed either no (2 of 5) or smaller tumor (3 of 5) within 24 days (Fig. 3g) when compared to siControl group. In addition, mice injected with siRASAL2 had significantly reduced mean tumor size (*P* < 0.01) and mean tumor weight (*P* < 0.01). shRNA stable knockdown system was also applied to xenograft mouse model and the tumorigenic ability of the gene was confirmed (Additional file 2: Figure S4).

### Ectopic expression of RASAL2 promoted tumorigenesis in CRC

**Fig. 4:**
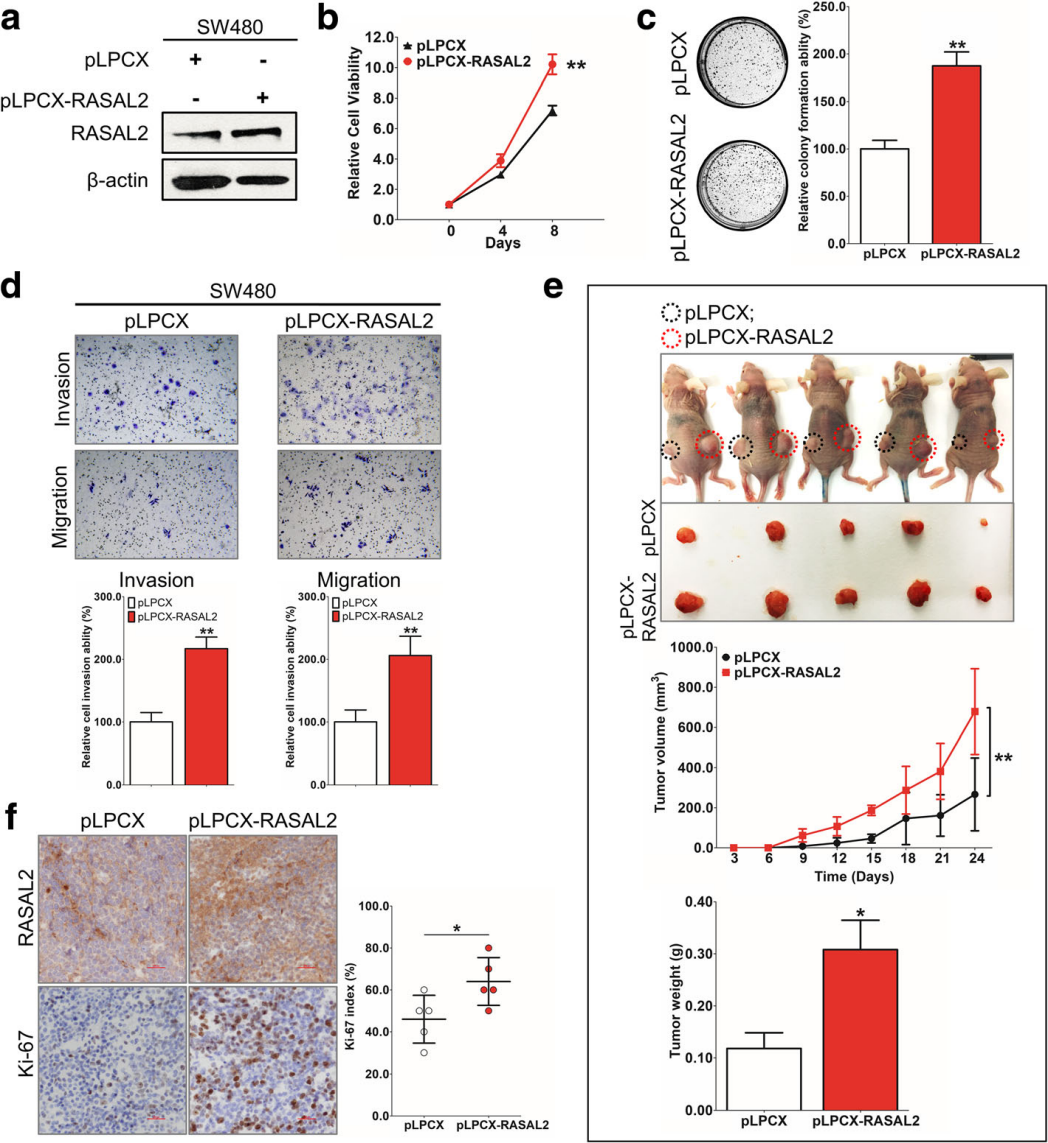
Ectopic expression of RASAL2 promotes CRC tumorigenesis. **a** Ectopic stable expression of RASAL2 protein in SW480 confirmed by western blot. **b**-**d** Ectopic expression of RASAL2 significantly enhanced cell viability **b**, anchorage-dependent growth (**c**) as well as cell invasion and migration (**d**) by MTT, colony formation and transwell cell invasion and migration assays in SW480 cells stably overexpressing RASAL2. **e** Representative image of tumors in xenograft mouse model (Left: pLPCX; Right: pLPCX-RASAL2) and that of tumor formed in nude mice inoculated subcutaneously with RASAL2 plasmids or empty vectors (Upper panel); The tumor growth curve of SW480 stably transduced with RASAL2 in nude mice was significantly dampened compared with SW480 transduced control (Middle panel); The mean tumor weights in the pLPCX-RASAL2 and pLPCX vector groups were summarized in histogram (Lower panel). **f** IHC assay of RASAL2 and Ki-67 in xenograft tumor tissues. (*, *P* < 0.05; **, *P* < 0.01, Student’s t-test, n = 3 independent experiments)

Gain-of-function study was performed by stably transfecting RASAL2 expression vectors (empty vector pLPCX and pLPCX-RASAL2 plasmid) into SW480 cells. Ectopic expression of RASAL2 in these cells, as shown by western blot (Fig. 4a), caused a significant increase in cell viability in SW480 compared to empty vector transfected cells (*P* < 0.01, Fig. 4b). RASAL2-overexpressing cells also showed increased colony formation (1617.0 ± 72.7) compared to cells receiving empty vector (1217.0 ± 44.1) (*P* < 0.01, Fig. 4c). Moreover, the invasion and migration abilities enhanced significantly in SW480 cells stably expressing RASAL2 (Fig. 4d) compared to those without. In nude mice, SW480-pLPCX-RASAL2 had significantly shorter latency of tumor formation, larger tumor size (*P* < 0.01) and larger mean tumor weight (*P* < 0.05) than SW480-pLPCX (Fig. 4e). SW480 xenografts overexpressing RASAL2 showed increased cell proliferation by Ki-67 staining (*P* < 0.05, Fig. 4f).

### RASAL2 is involved in the hippo signaling pathway

**Fig. 5:**
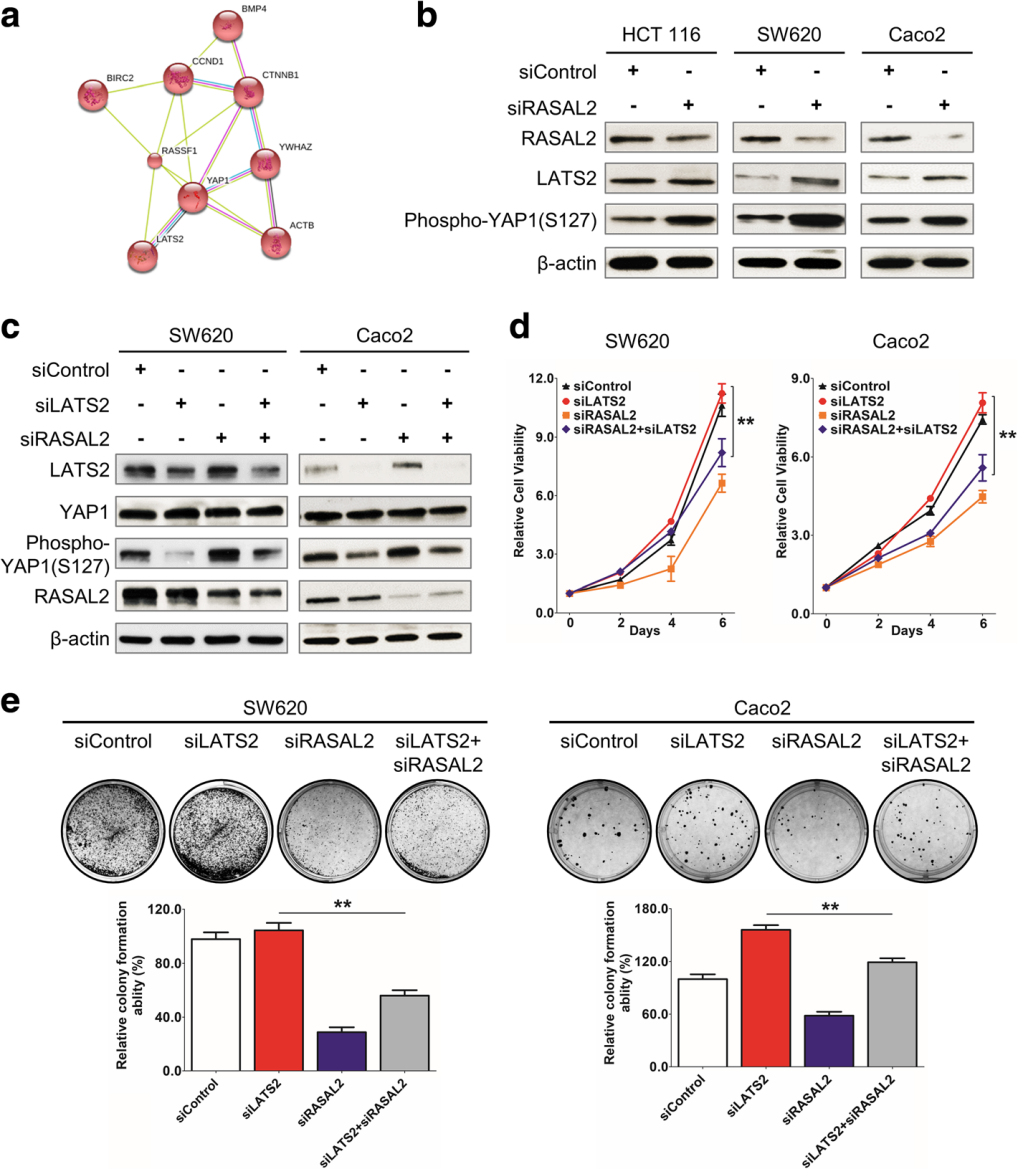
RASAL2 is involved in hippo pathway. **a** KEGG pathway enrichment analysis of RASAL2-regulated hippo pathway genes. **b** Western blot results showed that a higher level of LATS2 and phospho-YAP1 (Ser127) in siRASAL2 cells than that in the siControl group. **c** Western blot of LATS2, YAP1 and phospho-YAP1 (Ser127) expression upon LATS2 and RASAL2 double knockdown in CRC cells. Treatments with siControl, siRASAL2 and siLATS2 were shown in the upper panel. Phospho-YAP1 (Ser127) in LATS2 and RASAL2 double-knockdown group was high in siLATS2 group, but lower than that of the siRASAL2 group. **d** MTT assay showed siRNA-mediated double knockdown of RASAL2/LATS2 inhibited cell growth compared to the siLATS2 group in SW620 and Caco2 cells. **e** Colony formation assay showed reduced cell colonies in siRASAL2/siLATS2 double-knockdown cells, when compared to the siLATS2-treated cells. (*, *P* < 0.05; **, *P* < 0.01, Student’s t-test, n = 3 independent experiments)

To elucidate the molecular mechanism by which RASAL2 promotes development and progression in CRC, expression of RASAL2-modulated target genes in CRC cells transfected with siRASAL2 was compared to that in CRC cells transfected with control siRNA using Illumina HumanHT-12 Expression BeadChip arrays. Successful knockdown of RASAL2 by siRNA was confirmed using qRT-PCR and the efficiency ranged from 13.3 to 42.8% (Additional file 2: Figure S5). KEGG pathway enrichment analysis by STRING [32, 33] showed that the hippo signaling pathway was significantly enriched with differentially expressed genes (Fig. 5a). We confirmed the induction of LATS2 and YAP1 phosphorylation upon siRASAL2 treatment in a panel of CRC cell lines with or without *KRAS/NRAS* mutation (Fig. 5b). We next investigated whether the oncogenic effect of RASAL2 is dependent on its ability to downregulate LATS2. Given that RASAL2 abrogated the tumor suppressor function of LATS2, we conducted rescue experiment in which we co-transfected siLATS2, siRASAL2 or both into SW620 (*KRAS* G12 V) and Caco2 (*KRAS/NRAS* wild-type) cells and evaluated the effect on phospho-YAP1(S127) expression. LAST2 depletion reduced phospho-YAP1 (Ser127) but the level of total YAP1 remained unchanged (Fig. 5c). Double knockdown of LATS2 and RASAL2 increased YAP1 phosphorylation when compared to siLATS2 treated cells but could not fully restored the normal effect of LATS2, suggesting that siRASAL2 could partially rescue the effect of YAP1 dephosphorylation caused by LATS2 depletion (siRASAL2/LATS2 vs. siLATS2, Fig. 5c). Also, cell proliferation rate (Fig. 5d) and anchorage dependent growth (Fig. 5e) in siRASAL2/siLATS2 double knockdown cells were significantly reduced when compared with siLATS2 cells. These findings showed that LATS2 downregulation underlies oncogenesis induced RASAL2 in CRC. From another perspective, we observed a significantly lower level of phosphor-YAP1 in siRASAL2/LATS2 double knockdown cells when compared to siRASAL2-treated cells. This suggested that kinases other than LATS2 might be involved in siRASAL2-mediated YAP1 phosphorylation.

### Signal transduction in the RASAL2-hippo cascade

**Fig. 6:**
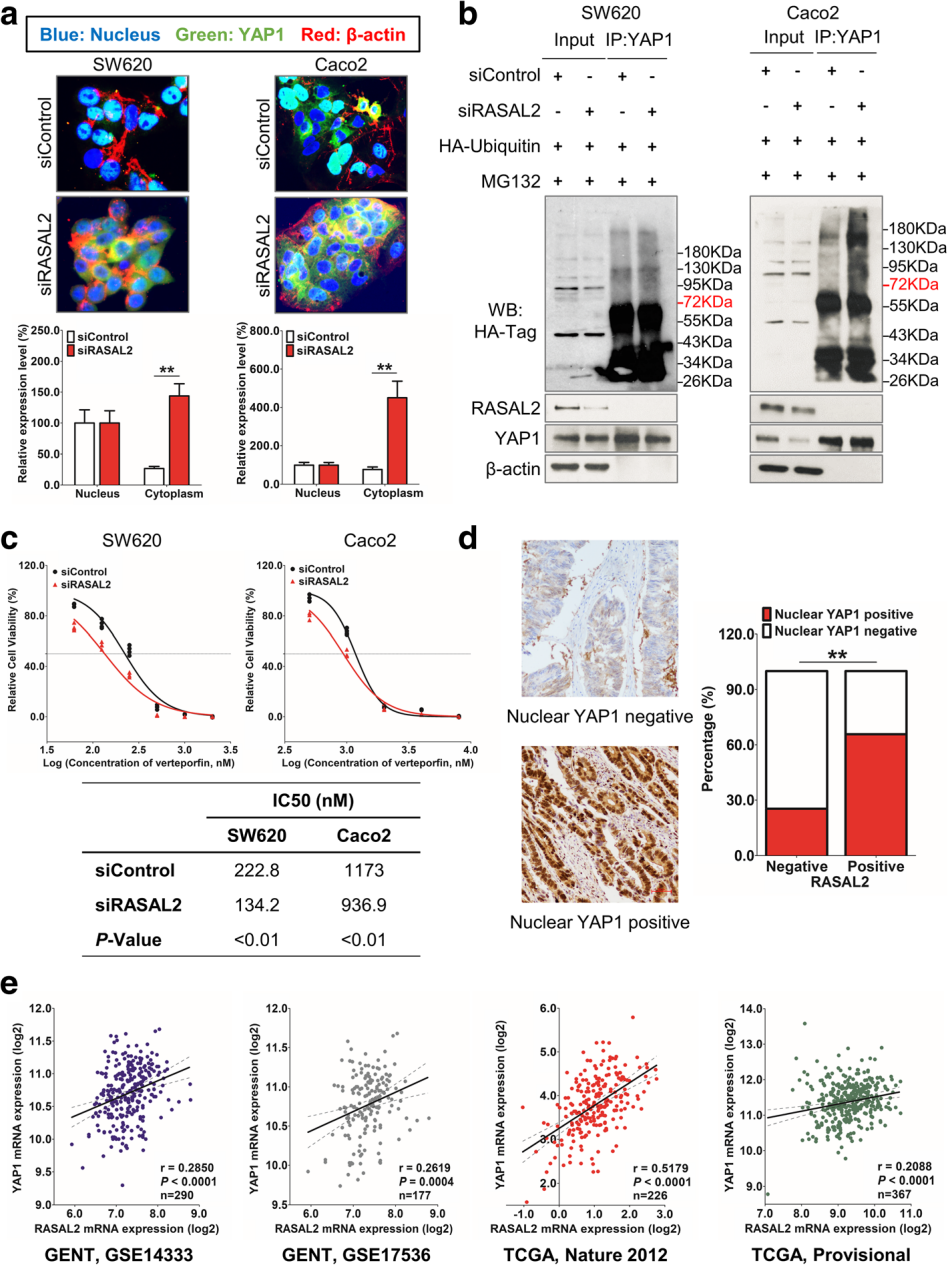
Signal transduction in the RASAL2-hippo cascade. **a** Knockdown of RASAL2 significantly increased translocation of YAP1 from the nucleus to cytoplasm in SW620 and Caco2 cells, Blue: Nucleus, Green: YAP1, Red: β-actin. **b** Immunoblotting with anti-HA antibody showed that more YAP1 proteins were ubiquitinated in siRASAL2 groups when compared to siControl in CRC cells, whereas proteasome inhibitor MG132 treatment lead to no further increase in YAP1 degradation through the proteasome. **c** IC50 of verteporfin decreased significantly in RASAL2 downregulated group when compared with siControl group in SW620 and Caco2 cells at 24 h after verteporfin incubation. **d** Nuclear YAP1 negative (Upper) and positive (Lower) using IHC of YAP1 in CRC; Nuclear YAP1 was overexpressed and significantly positively correlated with RASAL2 (Right, *P* < 0.01). **e** Overexpression of YAP1 was positively associated with RASAL2 mRNA expression in GENT and TCGA. (*, *P* < 0.05; **, *P* < 0.01, Student’s t-test, n = 3 independent experiments)

We then examined the subcellular localization of YAP1 upon siRASAL2 treatment in CRC cells by immunofluorescence. Knockdown of RASAL2 significantly increased translocation of YAP1 from nucleus to cytoplasm in SW620 (*KRAS* G12 V) and Caco2 (*KRAS/NRAS* wild type) cells (Fig. 6a). Since YAP1 phosphorylation promotes its binding to 14–3-3, cytoplasmic retention as well as subsequent poly-ubiquitination and degradation, we tested whether YAP1 ubiquitination could be stimulated by siRASAL2. As shown in Fig. 6b, YAP1 ubiquitination was elevated when SW620 and Caco2 cells were treated with RASAL2 siRNA, and this is consistent with the situation under which the hippo signaling pathway was turned on. To further delineate the effect of the pathway on RASAL2-mediated tumorigenesis, the pharmacological YAP1 inhibitor verteporfin, which is a small molecule inhibiting TEAD-YAP1 interaction [34], was introduced into SW620 and Caco2. The IC50 of verteporfin was 222.8 nM in SW620-siControl cells but decreased to 134.2 nM when treated with siRASAL2 at 24 h after verteporfin incubation (Fig. 6c). The results in Caco2 cells were similar. To evaluate the correlation of RASAL2 and YAP1 expression in clinical specimens, IHC of YAP1 was performed in 208 CRC samples and 42.3% samples had YAP1 protein detected in the nucleus. Moreover, YAP1 expression was positively correlated with RASAL2 expression in these samples (*R* = 0.403, *P* < 0.01, Fig. 6d), and the same results were observed in GENT [26] (GSE14333 [35], GSE17536 [36]) and TCGA [29, 30] (Fig. 6e).

**Fig. 7:**
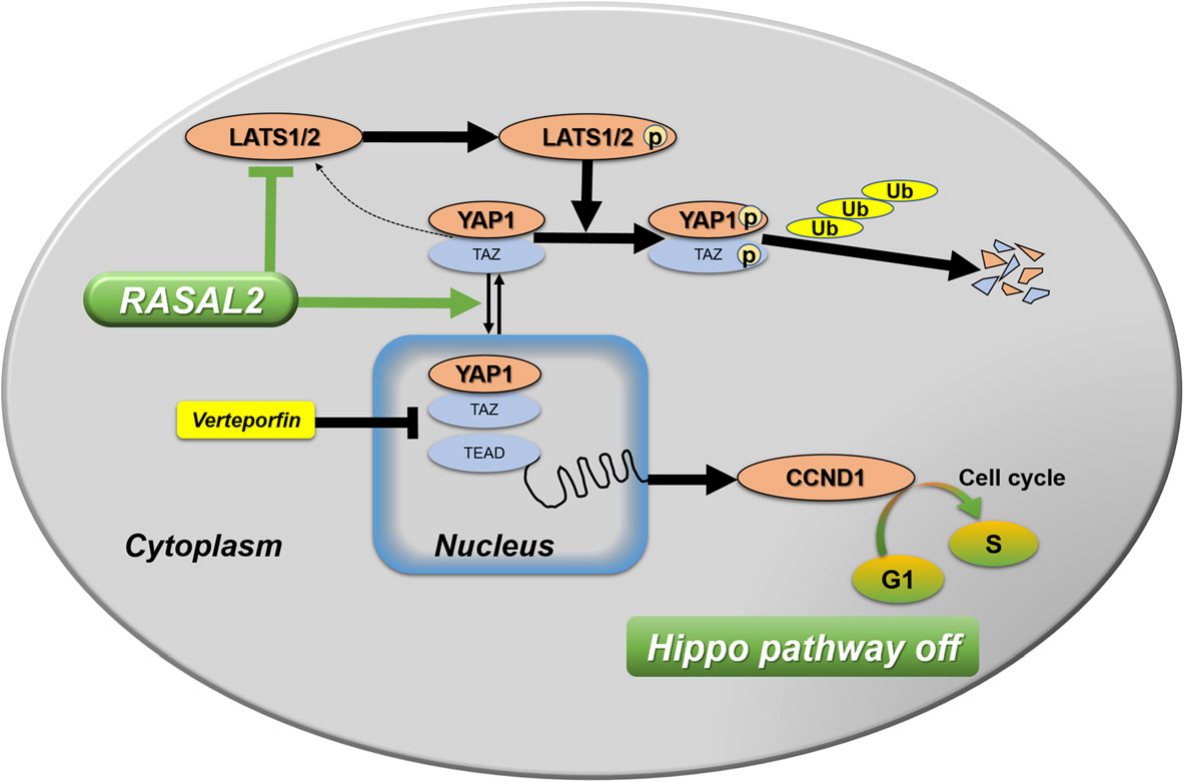
Outline of RASAL2-mediated hippo signaling pathway in CRC. RASAL2 showed high expression in CRC and targeted LATS2/YAP1 axis. This led to YAP1 dephosphorylation and nuclear translocation, thus preventing YAP1 from ubiquitination in the cytoplasm and functioning as a transcriptional co-activator to stimulate expression of pro-proliferation genes like CCND1 in CRC

In summary, RASAL2 showed high expression and targeted LATS2/YAP1 axis in CRC. YAP1 was therefore dephosphorylated and translocated to the cell nucleus. This prevented YAP1 from ubiquitination in the cell cytoplasm and functioning as a transcriptional co-activator to stimulate expression of pro-proliferation genes like CCND1 in CRC (Fig. 7). Collectively, we demonstrated the oncogenic role of RASAL2 in promoting tumorigenesis as well as metastasis and revealed that RASAL2 exerted its oncogenic property through LATS2/YAP1 axis of hippo signaling pathway in CRC.

## Discussion

This study showed, for the first time, that RASAL2 overexpression in CRC exerted 2 major effects: tumorigenesis and metastasis. Also, RASAL2 predicted poor disease outcomes independently, and RASAL2 promoted colon carcinogenesis through LATS2/YAP1 axis of hippo signaling pathway.

The RAS pathway is one of the most deregulated signaling pathways in human cancer [37] . The RAS family proteins are small molecule GTPases that relay extracellular signals to intracellular effector pathways. The guanine nucleotide exchange factors (GEFs) and the GTPase activating proteins (GAPs) serve as two key modulators that regulate the activity of RAS GTPases [38] . So far a total of 14 RAS GAP genes have been identified in human genome [39]. RASAL2 was initially identified as a tumor suppressor [40] and the inactivation of RASAL2 promoted tumor progression and metastasis in luminal B breast, ovarian, and lung cancers [13, 15, 16]. However, other studies found that RASAL2 promoted mesenchymal invasion and metastasis [14, 17–19]. A genome-wide study revealed that RASAL2 depletion inhibits cancer cell growth and invasiveness in liver and triple-negative breast cancers [14, 17]. Feng et al [14] reported a pro-oncogenic role of RASAL2 in triple-negative breast cancer. Their studies demonstrated that RASAL2 promoted mesenchymal invasion and metastasis through activating RAC1 signaling pathway independent of its RAS GAP catalytic activity. They also showed that RASAL2 was a functional target of anti-invasive mircroRNA-203, and RASAL2 expression was associated with poor clinical outcome, early metastasis and disease recurrence in patients with triple-negative breast cancer [14]. This is similar to our finding that RASAL2 played an oncogenic role in CRC tumorigenesis in vitro and in vivo, regardless of *KRAS/NRAS* mutation status.

Our findings appear to contradict a previous finding that RASAL2 was a tumor suppressor whose downregulation resulted in increased tumor growth, progression and metastasis in CRC [41]. Jia et al found that RASAL2 gene expression was negatively correlated with International Federation of Gynecology and Obstetrics (FIGO) stage [41]. However, in other cohorts like TCGA and GENT, RASAL2 consistently showed upregulation in CRC, and that is consistent with our results. Also, RASAL2 copy number gain was found in other gastrointestinal cancers, such as gastric, liver and pancreas cancer, in TCGA.

We found that RASAL2 was involved in hippo pathway in both *KRAS/NRAS* mutant and wild-type CRC cell lines. The core kinase-signaling cassette and downstream effectors of mammalian hippo pathway are highly conserved [42], including MST 1/2, SAV1, LATS 1/2, YAP1 and TAZ [43]. Activated MST1/2 interacts with SAV1 via the SARAH domains presented on both proteins, leading to phosphorylation and activation of direct substrates LATS1/2 [44]. YAP1 is a negatively regulated downstream target of the hippo signaling pathway. The mammalian ortholog of hippo kinase LATS2 suppresses the oncogenic activity of YAP1 oncogene by promoting YAP1 (Ser127) phosphorylation and subsequent cytoplasmic retention. Most of the known genes involved in the hippo signaling pathway are deregulated in human cancers and their expression levels as well as activities are correlated with tumor development and progression. Expression studies of common hippo signaling pathway components in CRC were also reported [6, 7, 45]. But the mechanism by which the hippo pathway is regulated is largely unknown. One of our key findings was that we identified RASAL2 as a novel regulator of the hippo pathway in colorectal tumorigenesis. In our study, we extracted the gene activation signatures of LATS2 and YAP1 from microarray data and performed western blot in RASAL2-downregulated CRC cells. RASAL2 accelerates CRC cell growth through interaction with LATS2-YAP1. The interaction between RASAL2 and YAP1 led to YAP1 dephosphorylation and nuclear translocation, thus preventing YAP1 from ubiquitination in the cytoplasm and functioning as a transcriptional co-activator to stimulate expression of pro-proliferation genes like CCND1 in CRC.

YAP1, the gene that received the most attention in the hippo signaling pathway, was found to be upregulated in CRC. However, YAP1 under-expression has also been observed in breast [46] and CRC [47] before. YAP1 is present in the nucleus and cytoplasm. Thus, both expression level and intracellular location of YAP1 must be considered when it is used as a biomarker. Our study further provided evidence for the constitutive interaction between RASAL2 and nuclear YAP1 in human CRC samples by IHC, therefore targeting the LATS2/YAP1 axis via RASAL2 may be more effective for the treatment of CRC in clinical settings. Previous studies showed that nuclear YAP1 was independently predictive of poor prognosis in 1028 CRC patients [48], and we verified that nuclear YAP1 was a predictor for worse survival in our cohort of 208 CRC patients (Additional file 2: Figure S6). But unlike RASAL2, nuclear YAP1 expression could not independently predict prognosis by multivariate cox regression analysis for our CRC patients (*P* = 0.609, Additional file 1: Table S8).

In addition to its role in tumorigenesis, RASAL2 induced tumor invasion and metastasis. When comparing the RASAL2 mRNA expression levels among normal colon mucosa, primary tumors and metastatic tumors, we found that metastatic tumors showed the highest RASAL2 expression, followed by primary tumors, and then normal colon mucosa. We also found that overexpression of RASAL2 was significantly associated with advanced tumor stages (III/IV) as well as lymphatic and distant metastases of CRC patients. RASAL2 knockdown in CRC cell lines also inhibited cell migration and invasion properties in vitro. We revealed that RASAL2 did not disturb the epithelial-mesenchymal transition (EMT) expression in CRC. Similarly, Feng et al [14] found that RASAL2 regulated mesenchymal invasion, without affecting EMT. Nevertheless, RASAL2 was reported to regulate EMT process in lung [15] and ovarian cancers [16] previously. Given the complexity and heterogeneity of molecular cancer pathways, it is not so surprising that such paradoxes exist in human cancers.

## Conclusions

In summary, our study highlighted the importance of RASAL2 in promoting tumorigenesis and metastasis in CRC. We also revealed, for the first time, that RASAL2 is involved in hippo pathway through the LATS2/YAP1 axis in CRC. An enhanced understanding of the gene in colorectal tumorigenesis might help establish a prognosis based on the genetic characteristics of each CRC patient and lead to personalized treatment for CRC.

## Additional files

Additional file 1: **Table S1.** Patients’ information in this study. **Table S2.** Primers sequence for qPCR and & siRNA/shRNA information. **Table S3.** DNA plasmids. **Table S4.** Copy number gain of 1q specially in metastases. **Table S5.** Folder changes of RASAL2 in primary and metastatic tumors by expression microarray analysis. **Table S6.** Clinicopathological correlation of RASAL2 expression in CRC. **Table S7.** Univariate and multivariate cox regression analysis of clinicopathologic factors with overall survival in CRC patients. **Table S8.** Univariate and multivariate cox regression analysis of nuclear YAP1 with overall survival in CRC patients. (PDF 231 kb)

Additional file 2: **Figure S1.** Hierarchical clustering of Illumina expression array in human CRC samples. **Figure S2.** RASAL2 expression of CRC in TCGA cohort. **Figure S3.** Western blot analysis for G1-S transition promoter CyclinD1 in cells with RASAL2 knockdown. **Figure S4.** shRASAL2 inhibited tumor growth in nude mice growth in vivo. **Figure S5.** RASAL2 RNA expression by siRNA knock down in CRCs. **Figure S6.** Kaplan Meier survival analysis of nuclear YAP1 expression in CRC patients. (PDF 551 kb)

## Abbreviations

aCGH: Array comparative genomic hybridization
AEEC: Animal experimentation ethics committee
AJCC: American joint committee on cancer
CRC: Colorectal cancer
DAPI: 4′,6-diamidino-2-phenylindole
EMT: Epithelial-mesenchymal transition
F: Female
FIGO: International Federation of Gynecology and Obstetrics
GAPs: GTPase activating proteins
GEFs: Guanine nucleotide exchange factors
GENT: Gene expression across normal and tumor tissue
H-score: Histoscore
IC50: Half maximal inhibitory concentration
IHC: Immunohistochemistry
INT: P-iodonitrotetrazolium violet
KEGG: Kyoto Encyclopedia of Genes and Genomes
M: Male
mut: Mutation
qRT-PCR: Quantitative real-time polymerase chain reaction
RNA array: Illumina expression array
RT: Reverse transcription
TCGA: The cancer genome atlas
TNM: tumor-node-metastasis
wt: Wild type
