# Supplementary material for: RASAL2 promotes tumor progression through LATS2/YAP1 axis of hippo signaling pathway in colorectal cancer
Source: Mol Cancer. 2018 Jul 23;17:102. doi: 10.1186/s12943-018-0853-6 (PMC6057036; doi:10.1186/s12943-018-0853-6)
Supplement: Supplementary file 1 — Table S1. Patients’ information in this study. Table S2. Primers sequence for qPCR and & siRNA/shRNA information. Table S3. DNA plasmids. Table S4. Copy number gain of 1q specially in metastases. Table S5. Folder changes of RASAL2 in primary and metastatic tumors by expression microarray analysis. Table S6. Clinicopathological correlation of RASAL2 expression in CRC. Table S7. Univariate and multivariate cox regression analysis of clinicopathologic factors with overall survival in CRC patients. Table S8. Univariate and multivariate cox regression analysis of nuclear YAP1 with overall survival in CRC patients. (PDF 231 kb) [file 12943_2018_853_MOESM1_ESM.pdf]

Additional file 1

Table S1. Patients' information in this study

NO.	Age (year)	Gender	Histology	TNM Classification	Site of colon	Metastatic Site	*RAS/RAF* Mutation	MSI	Array-CGH	RNA array
1	64	M	Adenocarcinoma	T3N1M0	Rectum	Lung	wt	MSS	✓	✓
2	54	M	Adenocarcinoma	T3N1M0	Recto-Sigmoid	Liver	wt	MSS	✓	
3	56	F	Adenocarcinoma	T3N2M1	Right	Liver	wt	MSS	✓	
4	61	M	Adenocarcinoma	T3N2M0	Sigmoid	Liver	wt	MSS	✓	✓
5	40	F	Adenocarcinoma	T3N1M0	Rectum	Liver	mut	MSS	✓	✓
6	69	F	Adenocarcinoma	T2N0M0	Sigmoid	Liver	mut	MSI-low	✓	
8	63	F	Adenocarcinoma	T3N0M0	Sigmoid	Liver	mut	MSI-low	✓	✓
142	73	F	Adenocarcinoma	T3N2M0	Caecum	Liver	mut	MSS	✓	

Table S2. Primers sequence for qPCR and & siRNA/shRNA information

Gene	Sequence	Supplier
RASAL2	Sense: TGTTCTGTCCTTGAGCCAGT	IDT
	Anti-sense: TCCACCTCAGACATCACCAA	IDT
β-actin	Sense: CGCGAGAAGATGACCCAGAT	IDT
	Anti-sense: GTACGGCCAGAGGCGTACAG	IDT
RASAL2_siRNA_1	CAGGAGGACCTTCTATTCGGA	Qiagen
RASAL2_siRNA_2	CGGCGACTGGAG GAATATGAA	Qiagen
RASAL2_shRNA_1	AATAAAGAGGAGTTGGCTTGTGCCTTAGT	TG309931, Origene
RASAL2_shRNA_2	CTCTTATTGCCAAGGTCATTCAGAACCTG	TG309931, Origene
LATS2_siRNA	CTCCGCAAAGGGTACACTCAA	Qiagen

IDT, Coralville, IA, USA

Qiagen, Hilden, Germany

Origene, Rockville, MD, USA

Table S3. DNA plasmids

DNA plasmid name	Objective	Selectable marker	Restriction Enzyme cutting site	Primer	Sequence of Primer
pcDNA3.1(+)/RASAL2	Transient transfection	Ampicillin	EcoRV	Sense	CGTgatatcAGCTCTCTCCGTCGTCC
			XhoI	Anti-Sense	ATTctcgagTCAGCAGCTGCTG
pLPCX-RASAL2	Stable clone	Ampicillin and Puromycin	Xho1	Sense	ATTctcgagAGCTCTCTCCGTCGTCC
			Not1	Anti-Sense	ATTGcggccgCTCAGCAGCTGCTG
pLPCX-Firefly luciferase	Stable clone	Ampicillin and Puromycin	BglII	Sense	CCGagatctCACCATGGAAGACGCCAA
			HindIII	Anti-Sense	GGGaagcttTCATCACTCGAGCAATTT

Table S4. Copy number gain of 1q specially in metastases

Sample ID	chrom	arm	start. position	end. position
**Patient 8 Metastasis**	1	q	169229095	215258644
**Patient 8 Metastasis**	1	q	169229095	235592711
**Patient 8 Metastasis**	1	q	169229095	228351874
**Patient 5 Metastasis**	1	q	169229095	169241274
**Patient 5 Metastasis**	1	q	169229095	225134351
**Patient 4 Metastasis**	1	q	196711595	196780309
**Patient 2 Metastasis**	1	q	152569860	152586234
**Patient 142 Metastasis**	1	q	242081818	242185893

Table S5. Folder changes of RASAL2 in primary and metastatic tumor by expression microarray analysis.

Gene Symbol	Expression Array			
	T/N. fc	M/N. fc	M/T. fc	ACCESSION
RASAL2	1.23569	1.49516	1.209976	NM_170692.1

Table S6. Clinicopathological correlation of RASAL2 expression in CRC (n = 208, significant *P*-value in bold and Italic format)

	All (n=208)	RASAL2		*P*-value
		Positive	Negative	
**Male gender**	113 (54.3%)	42 (50.0%)	71 (57.3%)	0.302
**Age at operation (years, mean ± SD)**	67.1 ± 12.4	65.7 ±13.6	68.0 ± 11.4	0.187
**Location**				0.371
**Right colon**	63 (30.3%)	25 (29.8%)	38 (30.6%)	
**Left colon**	47 (22.6%)	23 (27.4%)	24 (19.4%)	
**Rectum**	98 (47.1%)	36 (42.9%)	62 (50.0%)	
**Size (cm, mean ± SD)**	4.4 ± 1.7	4.3 ± 1.7	4.5 ± 1.7	0.406
**AJCC stage**				***0.018***
**I**	18 (8.7%)	6 (7.1%)	12 (9.7%)	
**II**	63 (30.3%)	17 (20.2%)	46 (37.1%)	
**III**	60 (28.8%)	25 (29.8%)	35 (28.2%)	
**IV**	67 (32.2%)	36 (42.9%)	31 (25.0%)	
**T stage**				0.084
**T1**	5 (2.4%)	3 (3.6%)	2 (1.6%)	
**T2**	21 (10.1%)	10 (11.9%)	11 (8.9%)	
**T3**	135 (64.9%)	46 (54.8%)	89 (71.8%)	
**T4**	47 (22.6%)	25 (29.8%)	22 (17.7%)	
**N stage**				***0.013***
**N0**	95 (45.7%)	28 (33.3%)	67 (54.0%)	
**N1**	66 (31.7%)	33 (39.3%)	33 (26.6%)	
**N2**	47 (22.6%)	23 (27.4%)	24 (19.4%)	
**M stage**				***0.007***
**M0**	141 (67.8%)	48 (57.1%)	93 (75.0%)	
**M1**	67 (32.2%)	36 (42.9%)	31 (25.0%)	
**Pre-ops CEA (ng/mL, mean ± SD)**	124.2 ± 733.1	90.7 ± 337.1	146.9 ± 909.1	0.588

Table S7. Univariate and multivariate cox regression analysis of clinicopathologic factors with overall survival in CRC patients (n = 208, significant *P*-value in bold and Italic format).

**Overall survival**	**Univariate**			**Multivariate**		
	**HR**	**95% CI**	**P-value**	**HR**	**95% CI**	**P-value**
**Male gender**	1.228	0.863-1.747	0.253			
**Age at operation (years)**	1.001	0.986-1.016	0.926			
**Location (right colon *vs.* left colon/rectum)**	0.969	0.652-1.440	0.876			
**Size (cm)**	1.032	0.933-1.142	0.538			
**T stage (T4 *vs.* T1/T2/T3)**	2.871	1.957-4.212	***<0.001***	0.995	0.992-0.999	***0.012***
**N stage (N1/N2 *vs.* N0)**	3.243	2.200-4.782	***<0.001***	1.934	1.244-3.006	***0.003***
**M stage (M1 *vs.* M0)**	7.280	4.928-10.756	***<0.001***	5.515	3.408-8.927	***<0.001***
**Pre-ops CEA >10 ng/mL**	2.608	1.792-3.795	***<0.001***	1.267	0.838-1.917	0.262
**RASAL2, H-score > 100**	1.684	1.180-2.404	***0.004***	1.561	1.074-2.269	***0.02***

Table S8. Univariate and multivariate cox regression analysis of nuclear YAP1 with overall survival in CRC patients (significant *P*-value in bold and Italic format).

**Overall survival**	**Univariate**			**Multivariate**		
	**HR**	**95% CI**	***P*-value**	**HR**	**95% CI**	***P*-value**
**Male gender**	1.228	0.863-1.747	0.253			
**Age at operation (years)**	1.001	0.986-1.016	0.926			
**Location (right colon *vs.* left colon/rectum)**	0.969	0.652-1.440	0.876			
**Size (cm)**	1.032	0.933-1.142	0.538			
**T stage (T4 *vs.* T1/T2/T3)**	2.871	1.957-4.212	***<0.001***	0.996	0.992-1.000	***0.045***
**N stage (N1/N2 *vs.* N0)**	3.243	2.200-4.782	***<0.001***	1.058	1.016-1.102	***0.007***
**M stage (M1 *vs.* M0)**	7.280	4.928-10.756	***<0.001***	6.753	3.801-11.998	***<0.001***
**Pre-ops CEA >10 ng/mL**	2.608	1.792-3.795	***<0.001***	1.171	0.725-1.893	0.518
**RASAL2, H-score > 100**	1.684	1.180-2.404	***0.004***	1.563	1.004-2.433	***0.048***
**Nuclear YAP1 positive**	1.684	1.124-2.525	***0.012***	1.131	0.705-1.813	0.609
