# Supplementary material for: RASAL2 promotes tumor progression through LATS2/YAP1 axis of hippo signaling pathway in colorectal cancer
Source: Mol Cancer. 2018 Jul 23;17:102. doi: 10.1186/s12943-018-0853-6 (PMC6057036; doi:10.1186/s12943-018-0853-6)
Supplement: Supplementary file 2 — Figure S1. Hierarchical clustering of Illumina expression array in human CRC samples. Figure S2. RASAL2 expression of CRC in TCGA cohort. Figure S3. Western blot analysis for G1-S transition promoter CyclinD1 in cells with RASAL2 knockdown. Figure S4. shRASAL2 inhibited tumor growth in nude mice growth in vivo. Figure S5. RASAL2 RNA expression by siRNA knock down in CRCs. Figure S6. Kaplan Meier survival analysis of nuclear YAP1 expression in CRC patients. (PDF 551 kb) [file 12943_2018_853_MOESM2_ESM.pdf]

Additional file 2

Figure S1

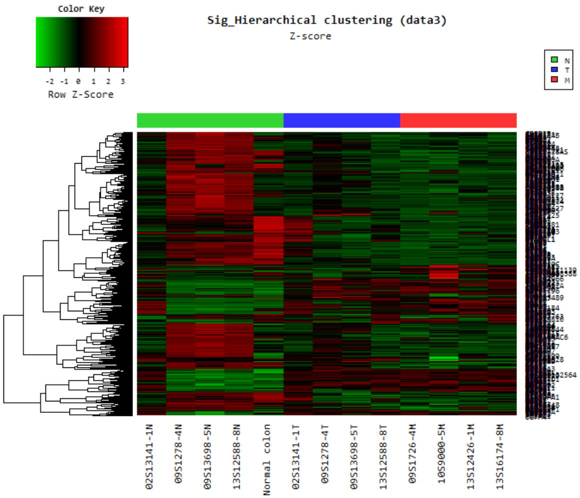

Figure S1 Hierarchical clustering of Illumina expression array in human CRC samples.

Figure S2

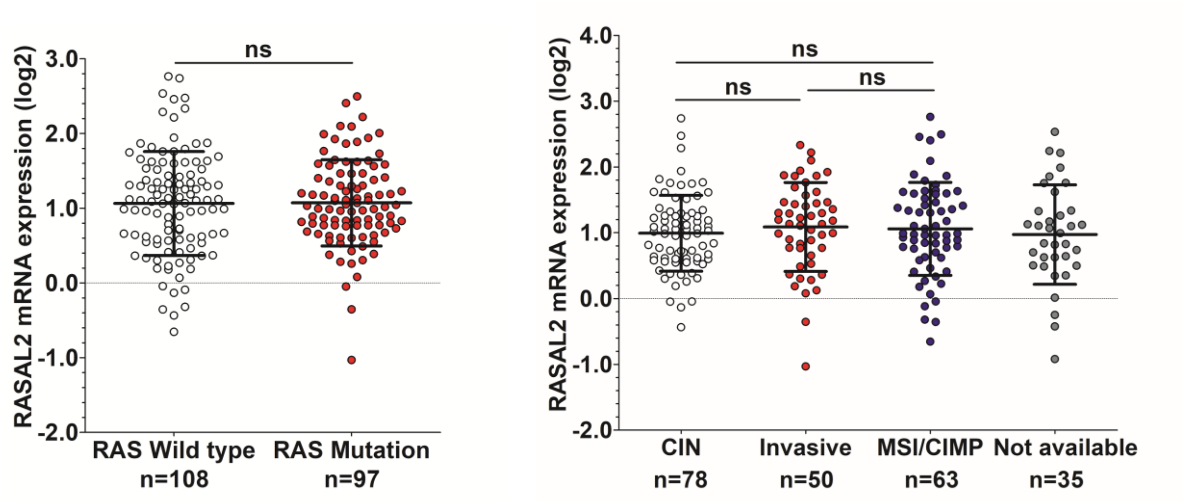

Figure S2 RASAL2 expression of CRC in TCGA cohort. Distribution of RASAL2 mRNA expression in *KRAS/NRAS* wild type and mutated tumors and in four gene expression subtypes of CRC for TCGA cohort (Nature 2012). ns: no significant.

Figure S3

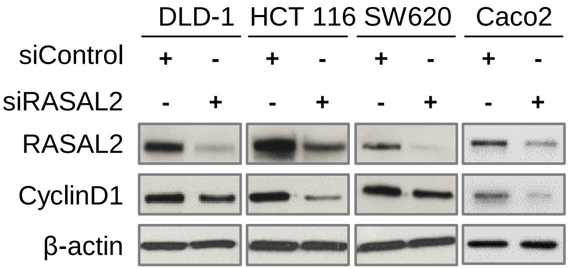

Figure S3 Western blot analysis for CyclinD1 in cells with RASAL2 knockdown. Western blot analysis confirmed that siRASAL2 suppressed the G1-S transition promoter cyclin D1, confirming the effect of siRASAL2 in blocking the cell cycle at the G1/S checkpoint.

Figure S4

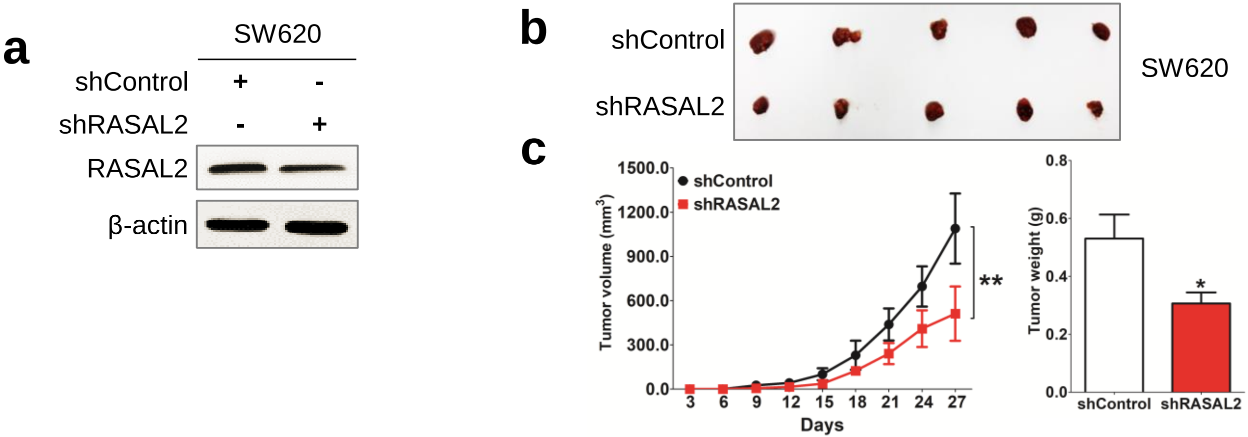

Figure S4 shRASAL2 inhibited tumor growth in nude mice growth in vivo. **a** Knockdown efficiency of RASAL2 in stable shRASAL2 downregulated SW620 cells was demonstrated by western blot. **b** A representative picture of tumor formation in nude mice subcutaneous inoculated with shRASAL2 or Control. **c** The tumor growth curve (Left) and tumor weight (Right) of SW620 stably transduced with shRASAL2 in nude mice was significantly dampened compared with Control cells. $^*P < 0.05$; $^{**}P < 0.01$.

Figure S5

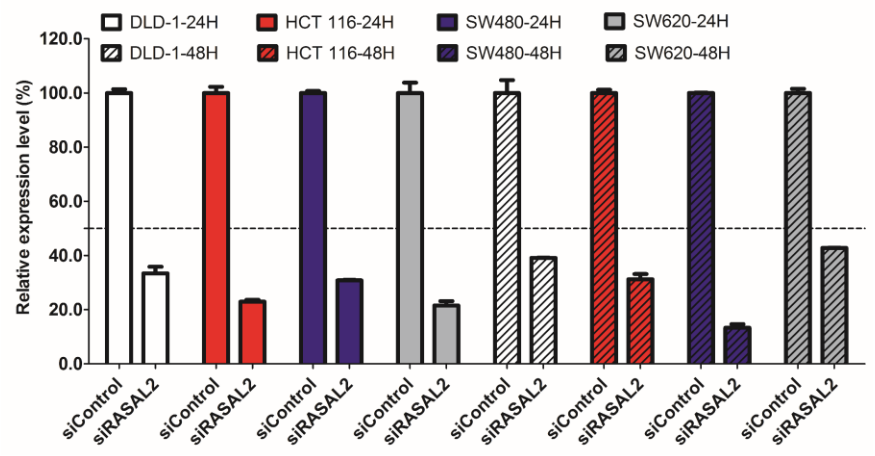

Figure S5 RASAL2 RNA expression by siRNA knockdown in CRCs. Successful knockdown of RASAL2 by siRNA was confirmed by qRT-PCR and the knock down efficiency ranged from 13.3% to 42.8%. β-actin was used as internal control.

Figure S6

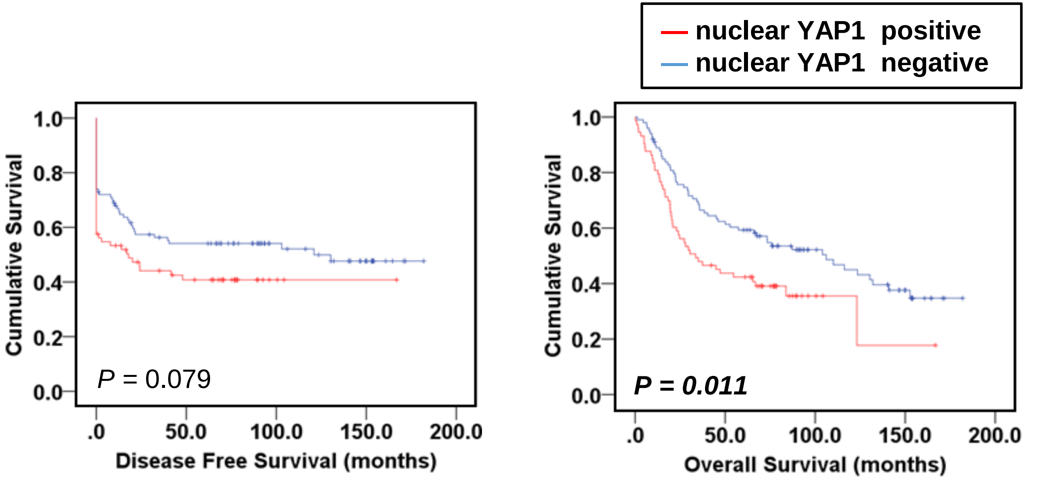

Figure S6 Kaplan-Meier survival analysis of nuclear YAP1 expression in CRC patients. Kaplan-Meier curve showed upregulation of nuclear YAP1 predicted significantly poorer overall survival in the local CRC patients (*P* = 0.011).
